# The spatial pattern and influencing factors of tourism eco-efficiency in Inner Mongolia, China

**DOI:** 10.3389/fpubh.2022.1072959

**Published:** 2022-12-13

**Authors:** Yuewei Wang, Xinyang Wu

**Affiliations:** ^1^School of Business, Liaoning University, Shenyang, China; ^2^School of Economics and Management, Northwest University, Xi'an, China

**Keywords:** tourism eco-efficiency, spatial evolution, influencing factors, Inner Mongolia, sustainable development

## Abstract

**Background:**

Tourism eco-efficiency is a performance basis for evaluating green total factor productivity and sustainable development.

**Objective:**

The objective of this study was to measure tourism eco-efficiency in Inner Mongolia and explore its influencing factors. The aim was to provide an accurate reference for improving the quality and efficiency of tourism in Inner Mongolia and promoting the sustainable development of the regional economy and society.

**Methods:**

Tourism eco-efficiency in Inner Mongolia from 2009 to 2019 was calculated using a super-slacks-based measure (SBM) model with an undesirable output. The spatial variation function was used to explore the spatial evolution pattern of tourism eco-efficiency in Inner Mongolia, and the influencing factors of the spatial evolution were analyzed by geographically weighted regression.

**Results:**

Tourism eco-efficiency in Inner Mongolia is relatively low. Eco-efficiency values among cities in Inner Mongolia vary, and their distribution is not balanced. The structural eco-efficiency of tourism in Inner Mongolia has been consistent from 2009 to 2019. The degree of homogenization in the overall direction is relatively good. Furthermore, its spatial distribution form and internal structure evolution show a certain regularity and continuity. The pattern evolution of tourism eco-efficiency in Inner Mongolia is jointly driven by the economic level, environmental regulation, industrial structure, traffic conditions, resource endowment, and tourism reception facilities. These influencing factors show obvious spatial heterogeneity.

**Conclusion:**

From the perspective of Inner Mongolia, the difference in the tourism eco-efficiency value from 2009 to 2019 was relatively large, but the number of effective areas in the efficiency frontier generally showed a fluctuating growth trend. The range parameters of tourism eco-efficiency showed a decreasing trend, and the spatial correlation effect of tourism eco-efficiency in Inner Mongolia showed a decreasing trend under the influence of structural and spatial differentiation.

## Introduction

Tourism is one of China's strategic pillar industries. In addition to helping regional economic growth and poverty alleviation, tourism contributes significantly to aesthetics and ecological civilization in China (1). In recent perspectives, the tourism industry is growing at a fast pace, which has resulted in extensive industrial development, ecological damage, and environmental pollution (2). With the rapid development of the tourism economy, the impact of carbon emissions generated by tourism activities on the environment is expanding annually and smokeless industries have ceased to exist. This necessitates consideration of the environmental problems caused by the development of tourism (3). Sustainable tourism was first proposed by the International Conference on Sustainable Development in 1990 and basically advocates for consideration of the ecological environment while promoting economic development and increased attention to collateral environmental effects during the development of the regional tourism economy to allow for the sustainability of the tourism industry. The evaluation of tourism eco-efficiency is a feasible method to measure the sustainable development of tourism, and an important research method is to start with tourism eco-efficiency (4). Therefore, this study, which is based on tourism eco-efficiency, can provide a reference to optimize the allocation of tourism elements and improve the use of tourism resources. Furthermore, it can provide a reference to promote the quality and efficiency development of tourism and practice sustainable tourism (5). Tourism eco-efficiency is a principal indicator to determine the sustainable development of tourism; it considers the ecological environment while meeting the tourism demands. Tourism eco-efficiency can be used to evaluate the sustainable development of tourism in a relatively scientific manner because it summarizes industrial, economic, and environmental indicators (6).

Schaltegger first proposed the concept of eco-efficiency in 1990 (7), and then the World Business Council for Sustainable Development proposed a method for measuring the ratio of eco-efficiency (the ratio of the economics of a product or service to its environmental impact) (8). Afterward, they explored its efficiency in industries such as agriculture, forestry, and the service industry. Tourism eco-efficiency is derived from ecological efficiency. Due to the continuous enrichment of tourism products, the types of tourism activities are increasing, and the tourism economy is developing rapidly; therefore, the negative impact of tourism on the environment is gradually emerging. For example, the surge in the number of tourists will lead to increased carbon emissions in tourism destinations. The energy consumed by tourists and the solid waste generated will cause different degrees of damage to the ecological environment (9, 10). Gössling proposed the concept of tourism eco-efficiency in 2005 (11). Since then, an upsurge in research on tourism eco-efficiency has been observed. It is mainly developed from five aspects: concept definition (12), model construction (13), level measurement (14, 15), mechanism of action (16, 17), and countermeasures (18). In terms of research content, scholars mainly regard the estimation of carbon emissions as the core content of eco-efficiency measurement (19–22). For example, Guo analyzed the spatial pattern of provincial tourism eco-efficiency under the constraints of energy conservation and emission reduction (23). Wang studied the spatial evolution of the tourism eco-efficiency industry and the impact environmental regulations had on the industry (24). Huang explored the monitoring and evaluation of carbon footprint and ecotourism in Wuyuan County (25).

There are two main types of calculation methods for tourism eco-efficiency: single index and model methods (26–29). Because existing statistical data do not include carbon emissions attributed to tourism, the single index measurement of tourism eco-efficiency should be determined using the tourism peeling coefficient model based on carbon emissions from other industries. That is, the ratio of the environmental impact index and the tourism economic index is used to express tourism eco-efficiency. Liu used the single ratio method to calculate tourism eco-efficiency and compared the differences between provinces (30). Li used the single index to calculate tourism eco-efficiency and analyzed its consistent relationship with regional ecological security (31). However, the eco-efficiency measurement of the single index method was slightly inaccurate due to the limited selection of variables. Many scholars prefer the multi-index method of measurement, which is mostly based on the input–output model and calculates tourism eco-efficiency by means of data envelopment analysis (DEA) [super-DEA, super-slacks-based measure (SBM)] and other methods. Lu used the super-SBM model with an undesirable output to calculate tourism eco-efficiency, and used the Tobit model to analyze influencing factors (32). Li measured tourism eco-efficiency in Wuling Mountain using the DEA method and analyzed its spatial pattern and influencing factors *via* exploratory spatial data analysis (33).

From the perspective of eco-environmental protection, studies on tourism eco-efficiency are in line with China's goal of constructing an ecological civilization and high-quality economic development. This is of great significance for the sustainable development of tourism (2). Located in northern China, Inner Mongolia is an important ecological security barrier. Inner Mongolia is also one of the provinces with relatively rich grassland and forest resources in China. It is particularly important for safeguarding China's ecological security and constructing an ecological civilization (34, 35). However, the rapid economic growth of Inner Mongolia mainly depends on energy, metallurgy, and other resource-based industries, which has caused great pollution to the environment. Thus, the ecological environment of Inner Mongolia needs urgent improvement (36). Also, the special geographical location, natural conditions, and industrial development mode make the ecosystem of Inner Mongolia very fragile. Therefore, it is urgent to provide countermeasures and suggestions for constructing an ecological civilization and the sustainable development of Inner Mongolia (37). In the face of the increasing energy consumption of tourism and the deterioration of the ecological environment, the evaluation of tourism eco-efficiency in Inner Mongolia can effectively reflect the relationship between the economic activities of tourism and its ecological environment. This will play a positive role in effectively dealing with the deterioration of the ecological environment caused by tourism development and promoting the construction of an ecological tourism civilization in Inner Mongolia (4, 10). This study measures tourism eco-efficiency in Inner Mongolia. The conclusions obtained not only help the government and tourism enterprises to effectively avoid the mismatch caused by blind investment and the loss of resources and environmental efficiency, but also provide countermeasures and suggestions for the government to make targeted tourism development planning, according to the temporal and spatial evolution of tourism eco-efficiency in Inner Mongolia (16, 17). In short, tourism eco-efficiency is the weather vane of green tourism development. The measurement of tourism eco-efficiency in Inner Mongolia can provide more scientific policies and guidance for the development of tourism in Inner Mongolia, so as to promote the coordinated and sustainable development of regional tourism (30, 31).

A sound ecological environment is the material basis for human survival and development, and also an important condition closely related to human health. A healthy urban physical environment is an important factor for the sustainable development of human settlements in the future. Research on tourism eco-efficiency in Inner Mongolia is helpful to provide a reference for the ecologically sustainable development of Inner Mongolia. This will not only promote Inner Mongolia to a resource-saving and environmentally-friendly society but also promote Inner Mongolia to implement a strict ecological and environmental protection system. The exploration of tourism eco-efficiency in Inner Mongolia is conducive to solving the prominent environmental problems affecting people's health and can provide a reference for the construction of ecological civilization, green development, and human healthy life.

Most of the studies focus on the concept of tourism eco-efficiency and the calculation of the tourism carbon footprint. Research on the measurement index of tourism eco-efficiency is lacking. In addition, there are only a few evaluations and spatial evolution analyses of provincial tourism eco-efficiency. To fill this research gap, this study aims to construct an index system to measure tourism eco-efficiency based on the eco-efficiency theory and the actual background of China. Therefore, this study evaluates tourism eco-efficiency in Inner Mongolia and analyzes the spatial pattern and influencing factors of tourism eco-efficiency of different cities in Inner Mongolia. The research questions of this study are as follows:

1. What indexes and models can measure tourism eco-efficiency in a relatively reasonable way?
2. What is the spatial pattern of tourism eco-efficiency in different regions of Inner Mongolia?
3. Are there spatial differences?
4. If there are spatial differences, what are their causal factors?
5. What are the rules of spatial distribution and evolution?

These issues reflect the empirical measurement of tourism eco-efficiency at different scales and the dynamic mechanism behind its temporal and spatial evolution. Research on tourism eco-efficiency is essential for the sustainable development of tourism in Inner Mongolia. To answer the abovementioned research questions, we established three subgoals.

First, this study established the evaluation index system of tourism eco-efficiency in Inner Mongolia based on previous studies. The super-SBM model with an undesirable output was used to calculate tourism eco-efficiency in Inner Mongolia.

Second, the spatial variation function was used to analyze temporal and spatial evolutionary features of tourism eco-efficiency in Inner Mongolia.

Third, the factors influencing tourism eco-efficiency in Inner Mongolia were assessed using the geographically weighted regression (GWR) method.

## Materials and methods

### Study area

The Inner Mongolia autonomous region is located in the northern region of China, across northeast, north, and northwest China, adjacent to Heilongjiang, Jilin, and eight other provinces, bordering Russia, Mongolia, located at 37.24–53.23°N, and 97.12–126.04°E. The entire region consists of nine cities and three leagues, and covers an area of 1.18 million km^2^, with abundant grasslands, forests, mountains, rivers, and deserts among other natural resources and Manchu and Mongolian culture, ethnic customs, border ports, and other human tourism resources. In recent years, Inner Mongolia has made a significant effort to create the brand image of “bright Inner Mongolia is in the north of the motherland.” In 2020, Inner Mongolia planned to promote epidemic prevention and control and cultural tourism, the year-round reception of domestic tourists and domestic tourism revenue reached 125 million people and 240.406 billion yuan (RMB), indicating that the development of tourism has developed well (38). Therefore, it is representative to research tourism eco-efficiency in Inner Mongolia. In terms of the ecological environment, Inner Mongolia has a superior resource endowment, vast territory, huge reserves of natural resources, and rich types. Inner Mongolia ranks first in China for grassland, forest area, and per capita arable land, and its reserves of rare earth metals rank first in the world. The ecological status of Inner Mongolia is not only related to the survival and development of the people of all ethnic groups in the region, but also to the ecological security of its neighboring areas. Therefore, protecting the ecological environment of Inner Mongolia is of great significance for its green and sustainable development (34). At the beginning of the twentieth century, the exploitation of non-renewable resources, such as coal and oil, and extensive development caused serious harm to the ecological environment of Inner Mongolia. However, with the effective implementation of ecological protection policies in Inner Mongolia in recent years, various ecological indicators have been restored, which have promoted the sustainable ecological development of Inner Mongolia (35). A good ecological environment is the material basis for human survival and development. It is also an important condition closely related to human health. A healthy urban physical environment is an important factor for the future sustainable development of human settlements. Eco-tourism in Inner Mongolia began with the development of tourism in the early 1980s. Relying on the rich eco-tourism resources, such as grasslands, deserts, forests, lakes, wetlands, wild animals, and plants, eco-tourism in Inner Mongolia has achieved rapid development. Eco-tourism is a form of tourism to protect the ecological environment, and its biggest characteristic is protection (36). Research on tourism eco-efficiency in Inner Mongolia not only promotes Inner Mongolia as a resource-saving and environmental-friendly society but also promotes the implementation of a strict eco-environmental protection system in Inner Mongolia (37).

### Data sources

Given the availability and integrity of data, this study of 12 union city in Inner Mongolia in 2009–2020 data analysis, data mainly comes from China city statistical yearbook from 2010 to 20120, Inner Mongolia statistical yearbook, or from Inner Mongolia ecological environment agency's website or with partially missing data interpolation processing.

### Index construction

Tourism eco-efficiency is a derivative of the concept of eco-efficiency applied to tourism, which refers to the use of a small environmental impact in the development of the tourism industry to obtain a high economic output. Based on the reference of the index systems reported by Wang (24) and Li (33) among other scholars, this study combines the available data on Inner Mongolia and the characteristics of the tourism industry (Table 1). The input of tourism products [composed of the sum of the number of star hotels, travel agencies, and weighted scenic spots (3A or above scenic spots)], labor input (the number of employees in the tertiary industry), and capital are considered as input indicators (investment in tourism fixed assets, that is, the ratio of the total tourism income to the gross national product (GNP) is used for conversion). The total tourism income (the domestic tourism revenue and inbound tourism revenue) and total tourism person-times (domestic tourism person-times and inbound tourism person-times) are considered as the expected output indicators. Wastewater and sulfur dioxide emissions from tourism are considered as undesirable output indicators (there are no statistical data on tourism carbon emissions at the present stage, so industrial wastewater and sulfur dioxide emissions are collected, and the ratio of tourism revenue to GNP is used for conversion measurement) (39, 40).

**Table 1:** The tourism eco-efficiency measurement index system of Inner Mongolia.

**Category**	**Index name**	**Index characterization**
Input indicators	Tourism products	The sum of star hotels, travel agencies, and weighted scenic spots
	Labor force	Number of workers in the tertiary industry
	capital	Investment in fixed assets for tourism
Desired output indicators	Total tourism Revenue	Domestic tourism revenue and inbound tourism revenue
	Total number of visits	Domestic tourism arrivals and inbound tourism arrivals
Undesired output indicators	Tourism wastewater discharge	Tourism as a percentage of industrial wastewater discharged
	Sulfur dioxide emissions from tourism	Tourism as a percentage of industrial sulfur dioxide emissions

### Research methods

#### Super-SBM model with an undesirable output

The SBM model proposed by Tone is an improvement of the traditional DEA model. It addresses radial and angular deviations and allows a more accurate assessment of the relationship between the input and output. Based on this, the effective ranking of decision-making units can be realized. The super-SBM model with an undesirable output was used to measure tourism eco-efficiency in Inner Mongolia (24) through the following formula:


$$\{\begin{array}{ll} \min p=\;(1-\frac{1}{m}\sum_{i\;=\;1}^{m}\frac{s_{i}^{-}}{x_{ik}})/\left[1+\frac{1}{q_{1}+q_{2}}(\sum_{r\;=\;1}^{q_{1}}\frac{s_{r}^{+}}{y_{rk}}+\sum_{r\;=\;1}^{q_{2}}\frac{s_{t}^{b-}}{y_{ik}})\right] & \;\\ \;s.t.x_{k}=X\text{λ}+s^{-},\;y_{k\;}=Y\text{λ}-s^{+},\;b_{k}=\;b\text{λ}+\;s^{b-} & \;\\ \text{ λ }\geq \;0,\;s_{i}^{-}\geq \;0,\;s_{r}^{+}\geq \;0,\;s_{i}^{b-}\geq \;0 & \; \end{array}$$

Where *p* is the efficiency; *m, q*_1_, and *q*_2_ are the number of indicators for inputs, desired outputs, and undesired outputs; *x*_*k*_,*y*_*k*_, and *b*_*k*_ are input, desired output, and undesired output variables; *x*_*ik*_,*y*_*rk*_ and *y*_*tk*_ are the elements of input and output vectors; *X, Y, b* are input–output matrices; and $s_{i}^{-},s_{r}^{+},$ and $s_{t}^{b-}$ are the slack variables of input, desired output, and undesired output; and λ indicates column vectors.

#### Spatial variation function

The spatial variation function proposed by the geostatistician Matheron can analyze the spatial correlation and heterogeneity of geographic variables. It can also reasonably and effectively analyze the spatial variation law and describe the spatial correlation between random fields and random processes (41–43). The formula is expressed as follows:


$$\begin{array}{l} \gamma \left(k\right)=\frac{1}{2N\left(k\right)}{\overset{N\left(k\right)}{\underset{i=1}{\sum }}\left[Y\left(x_{i}\right)-Y\left(x_{i}+k\right)\right]}^{2} \end{array}$$

*Y*(*x*_*i*_) and *Y*(*x*_*i*_+*k*) are the observed values *Y*(*x*) of the geographic variables at the points *x*_*i*_ and *x*_*i*_+*k*,*N*(*k*) are the sample sizes of the *k* segmentation distance.

#### Geographically weighted regression

Geographically weighted regression focuses on the local effects of spatial objects. Based on the principle of regression, it attempts to explore the relationship between spatial variables under the premise of considering the spatial correlation of the samples. Based on this, the spatial variation and driving factors of the research object are extended forward, and the characteristics and laws of spatial variation are analyzed (44–46). The formula is expressed as follows:


$$\begin{array}{l} y_{i}=\beta_{0}\left(u_{i}v_{i}\right)+\underset{k}{\sum }\beta_{k}\left(u_{i},v_{i}\right)x_{ik}+\varepsilon_{i} \end{array}$$

Where *y*_*i*_ refers to the global dependent variable; *x*_*ik*_ is the independent variable; (*u*_*i*_*v*_*i*_) is the coordinate of the capital city of the *i* region; β_*k*_(*u*_*i*_, *v*_*i*_) is the spatial unit value of the continuous function in the *i* region; ε_*i*_ is the random error term,β_0_ and β_*k*_are the parameters; and *k*is the number of regions.

## Results

### Measurement and overall characteristics of tourism eco-efficiency in Inner Mongolia

#### Tourism eco-efficiency in Inner Mongolia

**Figure 1:**
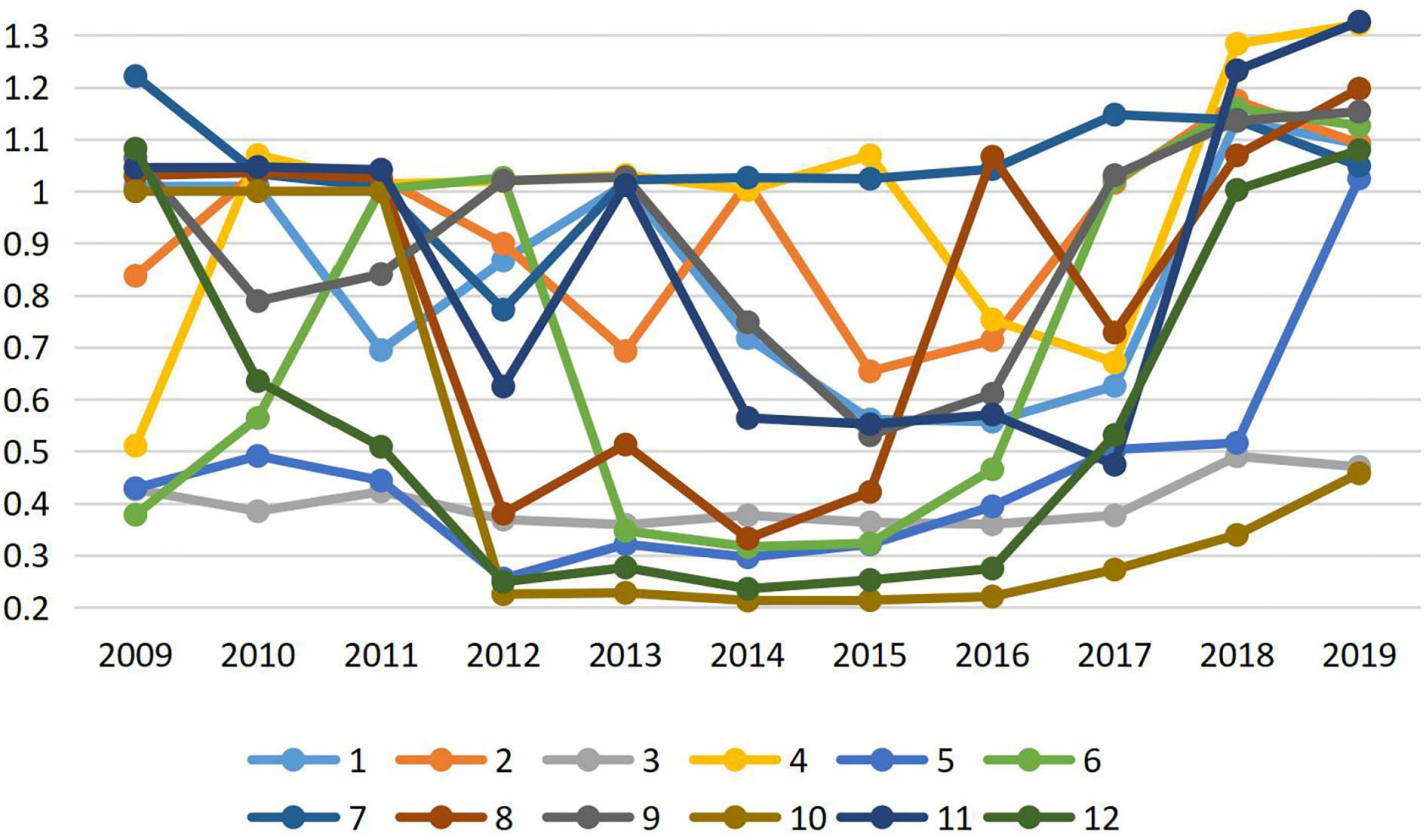
Tourism eco-efficiency in Inner Mongolia from 2009 to 2019. Hohhot City, Baotou City, Hulunbuir City, Xing‘an League, Tongliao City, Chifeng City, Xilin Gol League, Ulanqab City, Ordos City, Bayannur City, Wuhai City, and Alxa League are represented by numbers 1–12.

Based on the super-SBM model of the variable return scale (VRS) with an undesirable output, tourism eco-efficiency in Inner Mongolia from 2009 to 2019 was statically evaluated and obtained (Figure 1). Overall, tourism eco-efficiency in Inner Mongolia from 2009 to 2019 was generally low, with an average of only 0.74, indicating the presence of environmental pollution and resource waste in the tourism industry in Inner Mongolia, and there is a relatively wide scope to improve tourism eco-efficiency. From the perspective of Inner Mongolia, the difference in tourism eco-efficiency from 2009 to 2019 was relatively large, but the number of effective areas in the efficiency frontier generally showed fluctuating growth dynamics. From 2009 to 2019, the mean value of tourism eco-efficiency in Hohhot City, Baotou City, Ordos City, Hinggan League, and Xilin Gol League in Inner Mongolia reached the effective level, but tourism eco-efficiency in Alxa League, Wuhai City, Tongliao City, and Bayannur City was relatively low, and there was a significant margin of improvement. In short, tourism eco-efficiency in Inner Mongolia needs to be improved. The overall level of tourism eco-efficiency in Inner Mongolia is relatively low, and regional differences are relatively large. Tourism eco-efficiency in the central region of Inner Mongolia ranks first in the whole region. This is mainly due to the high level of economic development in the central region of Inner Mongolia, which has enough ability to invest in ecological construction and provides good conditions for its own green development.

#### Tourism eco-efficiency machine learning index

**Figure 2:**
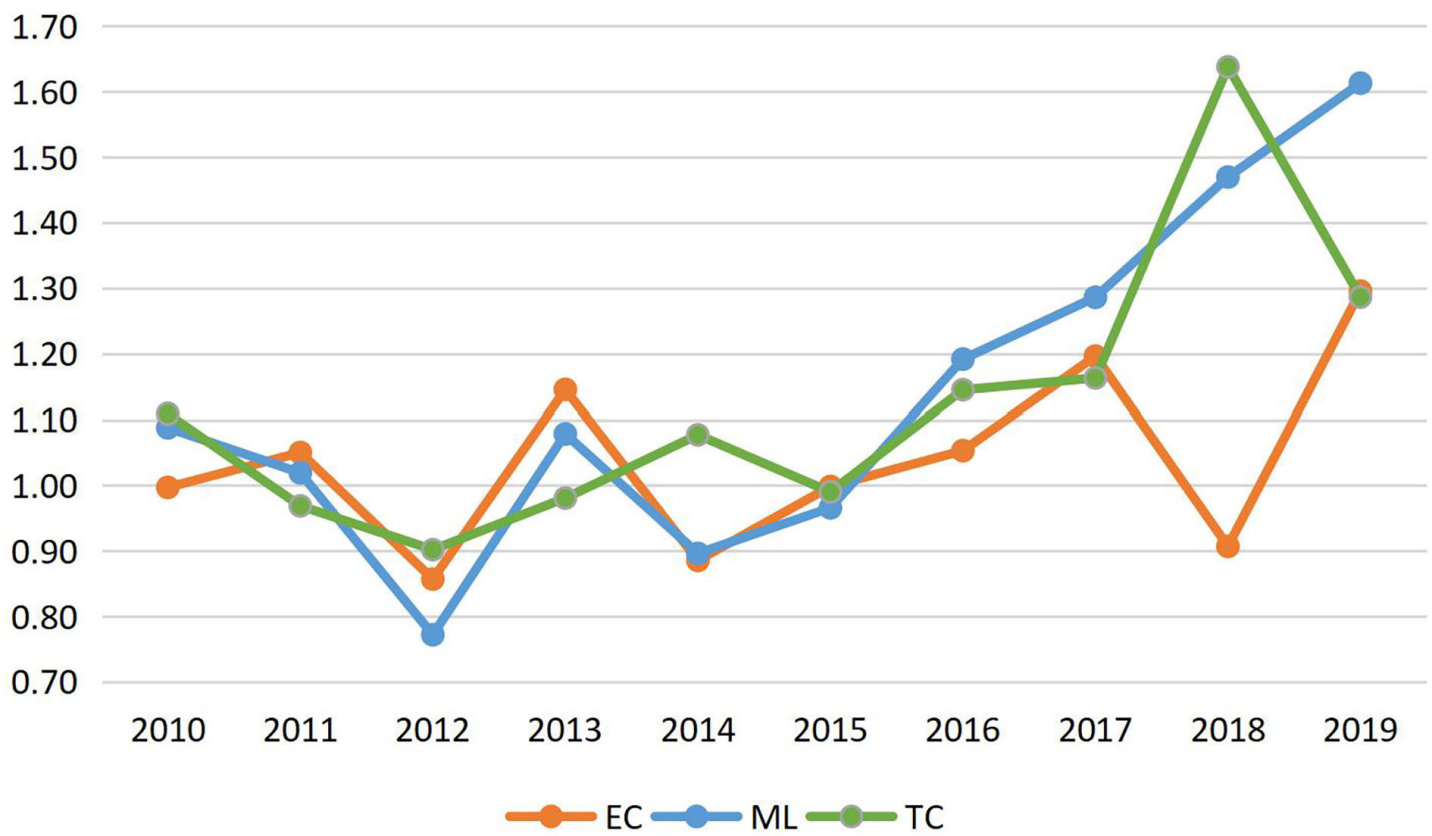
Tourism machine learning (ML) index and its decomposition in Inner Mongolia from 2009 to 2019.

Tourism eco-efficiency is a static measure that is independently measured yearly. Therefore, to determine the mobile changes in the tourism eco-efficiency levels in Inner Mongolia from 2009 to 2019, it is necessary to measure its growth rate. In this study, the VRS model was used for the measurement analysis considering the machine learning (ML) index, which refers to the growth rate of tourism eco-efficiency when the desired output is increased and the undesired output is reduced in equal proportion. The ML index includes two indicators: tourism eco-technical efficiency growth rate (EC) and tourism eco-technical progress growth rate (TC) (Figure 2). The ML index of an undesired output from 2009 to 2019 was greater than one in most years, indicating that tourism eco-efficiency in Inner Mongolia showed a trend of optimization. In terms of the average value of each year, the average growth rate of tourism eco-efficiency in Inner Mongolia is 13.80%, among which the contribution rate of technical efficiency and technological progress is 0.1 and 10.81%, respectively. The contribution rate of technological progress is significantly higher than that of technical efficiency, indicating that technological innovation plays an essential role in tourism eco-efficiency in Inner Mongolia. Due to a variety of factors, including the international green barrier, energy constraints, tightening in the “environmentally reversed transmission mechanism,” and the “five-sphere integrated plan” development philosophy, the government employs an active energy structure optimization strategy to boost capital investment, energy conservation, and emissions reduction. Technology research and development, as well as fiercely promoting technological innovation in its critical role in the development of the tourism industry, are all priorities.

### The spatial-temporal evolution of tourism eco-efficiency in Inner Mongolia

First, the projection coordinate system of Inner Mongolia was measured, then the spatial variation function of 2009, 2013, and 2019 Inner Mongolia tourism eco-efficiency was measured, and finally, the optimal model for measuring the fractal dimension of each direction was selected. Ultimately, the Kriging interpolation simulation was performed to comprehensively analyze the evolution process of the spatial pattern of tourism eco-efficiency in Inner Mongolia. According to the results of the spatial variation value of tourism eco-efficiency in Inner Mongolia (Table 2), the Gaussian model was selected as optimal for analysis. In addition, the structural features of tourism eco-efficiency in Inner Mongolia were consistent over the years, and its coefficient of determination tended to initially increase and then decrease, but overall it remained relatively stable. The range parameters of tourism eco-efficiency decreased from 2,809,386.41 m in 2009 to 1,293,841.95 m in 2019, indicating that the spatial correlation of tourism eco-efficiency in Inner Mongolia showed a narrowing trend under the influence of structural and spatial differentiation. Due to the vast territory and large east-west span of Inner Mongolia, there are huge differences in the economic foundation, resource endowment, infrastructure, and traffic conditions among the allied cities, which lead to limitations in their spatial correlation, core area radiation, and interregional spillover.

**Table 2:** Fitting parameters of the variation function of tourism eco-efficiency in Inner Mongolia.

**Year**	**Model**	**Co**	**Co+C**	**Co/(Co+C)**	**Range**	**R^2^**
2009	Gaussian	0.001000	0.540000	0.001851852	2809386.41	0.594
2013	Gaussian	0.027600	0.231200	0.119377163	1519008.56	0.374
2019	Gaussian	0.020300	0.253600	0.080047319	1293841.95	0.459

From the fractal dimension of the spatial variation function (Table 3), the overall direction of tourism eco-efficiency in Inner Mongolia exhibited a relatively good degree of homogenization, and the spatial difference in the overall direction fluctuated and increased, while the spatial difference in the local direction was relatively obvious. The dimension value in the overall direction increased from 1.420 in 2009 to 1.459 in 2019 but showed a transient decline in 2013. The south-north fractal dimension continued to increase, and the coefficient of determination was small and decreased continuously, indicating that the spatial difference in tourism eco-efficiency decreased in this direction, and the scale of differentiation was small and continuously decreased. The gap between tourism eco-efficiency in northern and southern Inner Mongolia is decreasing. The fractal dimension and the coefficient of determination in the northeast and southwest do not change significantly, indicating that the spatial difference and divergent scales of tourism eco-efficiency in this direction show a stable trend with little change and relatively balanced development. The fractal dimension increased from east to west, while the coefficient of determination shows a decreasing trend, indicating that the spatial difference in tourism eco-efficiency in this direction and the scale of differentiation tended to decrease. In short, the evolution of each direction exhibited unique properties. From the perspective of historical development, the eastern region of Inner Mongolia is dominated by the primary industry, while the western region has a certain first-mover advantage in the development of secondary and tertiary industries. However, the natural resources and rich cultural heritage of the east provide a prerequisite for the development of leisure tourism. All cities in Inner Mongolia pay attention to the development of tourism and the improvement of ecological efficiency. However, due to differences in the economic foundation, infrastructure, and the universality of policy coverage, the improvement of tourism eco-efficiency in Inner Mongolia is characterized by an overall improvement, but the local advantages are not obvious.

**Table 3:** The fractal dimension of the variation function of tourism eco-efficiency in Inner Mongolia.

**Year**	**Omnidirectional**		**N-S (0** **°** **)**		**EN-WS (45** **°** **)**		**E-W (90** **°** **)**	
	**D**	**R^2^**	**D**	**R^2^**	**D**	**R^2^**	**D**	**R^2^**
2009	1.420	0.620	1.091	0.229	1.521	0.670	1.174	0.652
2013	1.400	0.449	1.896	0.005	1.633	0.044	1.292	0.500
2019	1.459	0.339	1.868	0.006	1.537	0.089	1.990	0.000

**Figure 3:**
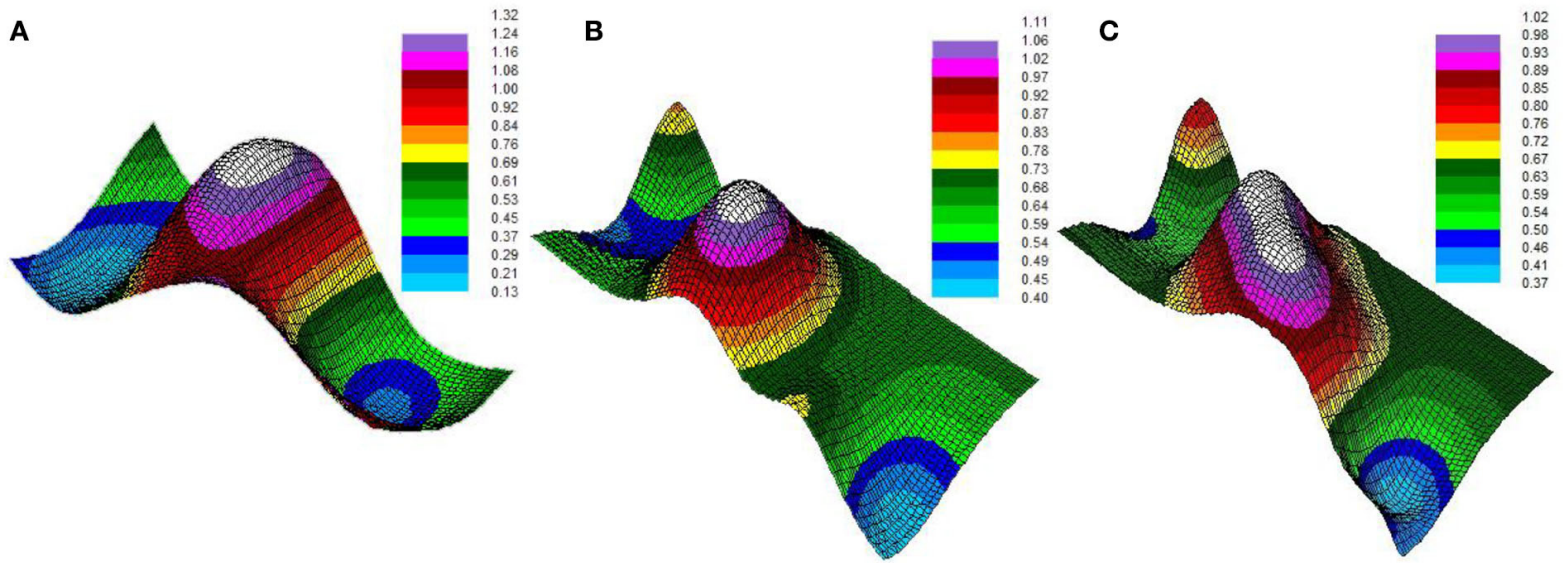
The Kriging interpolation simulation of the tourism eco-efficiency variation function in Inner Mongolia in 2009, 2013, and 2019. **(A)** 2009, **(B)** 2013, and **(C)** 2019.

The Kriging interpolation simulation of the variation function of tourism eco-efficiency in Mongolia (Figure 3) shows that the spatial distribution form and internal structure evolution of tourism eco-efficiency in Inner Mongolia have some regularity and continuity. In 2009, 2013, and 2019, the low-value areas of tourism eco-efficiency showed a trend of low-value dispersion and concentration, with Bayannur City and Alxa League being the low-value core and low eco-efficiency valley, respectively. At the same time, the high-value polar core areas were mainly focused on Hohhot City, Ulanqab City, and Xilin Gol League, and the siphon effect was the most significant. This is mainly due to the high level of economic development in the central region of Inner Mongolia, which has enough ability to invest in ecological construction and provides good conditions for its own green development. Due to geographical location, infrastructure, and other reasons, the eastern and western regions of Inner Mongolia have not achieved coordinated development of economic growth, environmental protection, and tourism development. Tourism eco-efficiency of the core cities in Inner Mongolia should have the positive effects of radiation and agglomeration and brings about the balance and coordinated development of the entire region.

### The influencing factors of tourism eco-efficiency in Inner Mongolia

Based on the objectivity of the spatial heterogeneity of the influencing factors of tourism eco-efficiency, the GWR method was used to measure the regression coefficients of the influencing factors in each region. Data from 2009, 2013, and 2019 were selected to construe the spatial evolution law of the influencing factors on the eco-efficiency level. Considering (*u*_*i*_, *v*_*i*_) as a *i* coordinate, the GWR model of tourism eco-efficiency is expressed as follows:


$$\begin{array}{l} TECO_{ij}=\beta_{0}\left(u_{i},v_{i}\right)+\beta_{1}\left(u_{i},v_{i}\right)pGDP_{y}+\beta_{2}\left(u_{i},v_{i}\right)TIP_{y}\\ +\beta_{3}\left(u_{i},v_{i}\right)CHU_{y}+\beta_{4}\left(u_{i},v_{i}\right)SWS_{ij}+\beta_{5}\left(u_{i},v_{i}\right)EPE_{ij}+\varepsilon_{ij} \end{array}$$

Where *TECO*_*ij*_is the tourism eco-efficiency level of the *i* region in the period *j*; *pGDP*_*ij*_, *TIP*_*ij*_, *SWS*_*ij*_, and *EPE*_*ij*_ represent the economic development (per-GDP), industrial structure (tertiary industry share), traffic condition (ratio of the length of graded highways to the urban area), resource endowment (the weighted score of high-level scenic spots), and environmental regulation (expenditure on energy conservation and environmental protection), respectively, in the period *J* of the region *i*.

ArcGIS10.4 was used to calculate GWR, and the regression coefficients of each influencing factor were divided into five levels ranging from the high-value area to the low-value area based on five levels of natural fracture points (Table 4). In terms of economic factors, from the perspective of space, tourism eco-efficiency in Hulunbuir City, Xing'an League, Tongliao City, and Chifeng City, among other cities was found to be greatly affected by the economic level, while tourism eco-efficiency in Bayannur City, Wuhai City, Alxa League, and other cities were less affected by the economic level. From the temporal perspective, the pattern of tourism eco-efficiency affected by the economic level of each year was basically the same. The spatial and temporal patterns of the impact of environmental regulation on tourism eco-efficiency were generally consistent with those of economic factors. Spatially, the tourism eco-efficiency factors in Wuhai City, Alxa League, and Bayannur City are greatly affected by industrial structure, while those in Bayannur City, Hulunbuir City, Xing‘an League City, Tongliao City, and Chifeng City and other tourism eco-efficiency factors are less affected by industrial structure factors. With respect to time, the pattern of tourism eco-efficiency affected by industrial structure in different years is basically the same; however, certain patterns exhibit slight variations. The spatial and temporal patterns of the influence of traffic conditions and resource endowment on tourism eco-efficiency are generally consistent with the spatial and temporal distribution pattern of the influencing factors of industrial structure. Summarily, tourism eco-efficiency in Inner Mongolia should be improved according to the heterogeneity of different countermeasures and suggestions.

**Table 4:** Natural fault zone division of each parameter in the geographically weighted regression (GWR) model.

	**Year**	**High-value area**	**Re-High-value area**	**Median value area**	**Re-Low-value area**	**Low-value area**
Economic development	2009	3,4,5	6,7	1,8	2,9,11	10,12
	2013	3	4,5,6,7	1,2,8	10	9,11,12
	2019	3,4,5	6,7	1,2,8	9,10	11,12
Industrial structure	2009	11,12	2,8,10	1,7,9	5,6	3,4
	2013	10,12	2,7,11	8,9	1,3,4	5,6
	2019	10,12	2,9,11	1,7,8	3,6	4,5
Traffic conditions	2009	10,12	2,9,11	1,8	7	3,4,5,6
	2013	12	2,9,10,11	1,8	3,7	4,5,6
	2019	12	9,10,11	1,2,8	6,7	3,4,5
Resource endowment	2009	10,12	2,11	1,8,9	7	3,4,5,6
	2013	9,11,12	1,2,10	8	5,6,7	3,4
	2019	11,12	1,9,10	2,8	6,7	3,4,5
Environmental regulation	2009	3,4,5,6	7	1,9	2,8,11	10,12
	2013	4,5,6	3	1,7,8	2,9,10,11	12
	2019	3,4,5	6,7	1,2,8	9,10,11	12

Hohhot City, Baotou City, Hulunbuir City, Xing'an League, Tongliao City, Chifeng City, Xilin Gol League, Ulanqab City, Ordos City, Bayannur City, Wuhai City, and Alxa League are represented by numbers 1–12.

## Discussion

This study uses a super-SBM model with an undesirable output to assess and analyze tourism eco-efficiency during 2009–2019 in Inner Mongolia. Then, using the spatial variation function analysis of the spatial and temporal evolution characteristics of tourism eco-efficiency in Inner Mongolia and based on the GWR analysis of the influence of factors of tourism in Inner Mongolia on eco-efficiency, the following conclusions were drawn.

The average tourism eco-efficiency in Inner Mongolia is 0.74, which is relatively low. Furthermore, the tourism eco-efficiency values of the provinces vary significantly, and their distribution is unbalanced. In addition, technological progress contributes significantly to the growth rate of tourism eco-efficiency in Inner Mongolia, indicating that technological innovation has a relatively high impact on tourism eco-efficiency. The differences in tourism eco-efficiency from 2009 to 2019 in Inner Mongolia were relatively large, but the number of effective areas in the efficiency frontier generally showed a fluctuating growth trend. The results of this study echo previous studies. Jun shows that the extensive economic growth mode restricts the improvement of tourism eco-efficiency in Inner Mongolia (47). The improvement of Inner Mongolia's technological level and the realization of scale efficiency are the fundamental ways to improve eco-efficiency and realize energy saving and emission reduction. On the whole, the value of tourism eco-efficiency in Inner Mongolia is generally low, indicating the occurrence of environmental pollution and resource waste in the development process of tourism in Inner Mongolia, and there is a relatively large room to improve tourism eco-efficiency (18, 32).

The range parameters of tourism eco-efficiency showed a decreasing trend, and the spatial correlation effect of tourism eco-efficiency in Inner Mongolia showed a decreasing trend under the influence of structural and spatial differentiation. Tourism eco-efficiency in Inner Mongolia showed consistent structural characteristics in different periods. The general homogenization degree of tourism eco-efficiency is relatively good, and the spatial difference in the local direction is relatively obvious. The spatial distribution pattern and internal structure evolution of tourism eco-efficiency have a certain regularity and continuity, showing a high-value concentrated distribution, and low-value scattered contiguous distribution. Yuanyuan and Yuxiang pointed out that the spatial variation function can deeply describe the randomness and structure of regional variables and measure the degree of variation of the spatial pattern of economic units (48). The structural characteristics of tourism eco-efficiency in Inner Mongolia are consistent over the years, and its coefficient of determination first increases and then decreases, but in general, it is relatively stable. The spatial difference in tourism eco-efficiency is decreasing from the south to the north, and the scale of differentiation is small and continuously decreasing. The gap between tourism eco-efficiency from the south to the north in Inner Mongolia is narrowing. The fractal dimension of tourism eco-efficiency in the east-west direction of Inner Mongolia shows an increasing trend, while the coefficient of determination shows a decreasing trend, indicating that the spatial difference of tourism eco-efficiency in Inner Mongolia is decreasing in this direction, and the scale of differentiation is smaller and continuously smaller. In short, the evolution of each direction has its own characteristics (41, 42).

The pattern evolution of tourism eco-efficiency in Inner Mongolia is jointly driven by the economic level, environmental regulation, industrial structure, traffic conditions, resource endowment, and tourism reception facilities, and there is obvious spatial heterogeneity among the influencing factors. The spatial and temporal patterns of the impact of environmental regulation on tourism eco-efficiency are generally consistent with those of economic factors. The spatial and temporal patterns of the influence of traffic conditions and resource endowment on tourism eco-efficiency are generally consistent with those of the influencing factors of industrial structure. Zhilong and Diyun emphasized that the economic level, industrial structure, resource endowment, infrastructure, and environmental regulation are the key factors affecting tourism eco-efficiency (33, 41). The regional economic level is closely related to the development of the tourism industry, which affects the development level of regional tourism to a certain extent. The optimization of the industrial structure is conducive to the healthy development of the tourism industry, thus affecting tourism eco-efficiency (44). Tourism resources are the foundation of tourism development, and resource endowment will inevitably have an important impact on tourism eco-efficiency. Infrastructure, such as traffic conditions, is an important objective condition for the smooth development of regional tourism activities, which will also have a certain impact on tourism eco-efficiency (45).

### Implications

This study first constructs a tourism eco-efficiency evaluation index system based on Inner Mongolia and then explores the evolutionary path and spatial pattern of tourism eco-efficiency in Inner Mongolia from the perspective of the geographic spatiotemporal dimension. To provide an accurate reference for improving tourism quality and eco-efficiency in Inner Mongolia and the sustainable development of the regional economy and society.

### Theoretical implications

First, the study presents research on tourism eco-efficiency from the perspective of ecological and environmental protection, which conforms to the connotation of developing an ecological civilization and meets the requirements of high-quality economic development. This is of great significance for enriching the theory of ecological civilization construction and expanding the applicable category of ecological civilization construction. Second, tourism eco-efficiency is the application of the theory of tourism eco-efficiency. Tourism eco-efficiency can combine the development of the tourism economy with its environmental impact, which can also provide some academic reference for the study of eco-efficiency in other industries. Finally, this study tries to determine the temporal evolution path and spatial pattern of tourism eco-efficiency, which is the basic paradigm of geographic spatiotemporal analysis. With respect to research, this study incorporated the undesired output in the tourism eco-efficiency measurement and constructed a relatively scientific, systematic, and perfect tourism eco-efficiency evaluation index system. In addition, the rules of spatiotemporal evolution and the characteristics of tourism eco-efficiency based on Inner Mongolia were analyzed. This provides additional insight into the correlation between geographical spatial patterns and the ecological environment, and promotes the cross-integration of tourism economics and tourism geography and other marginal disciplines. With respect to research methods, this study adopts the super-SBM model with the undesirable output to calculate tourism eco-efficiency in Inner Mongolia from 2009 to 2019, and comprehensively uses the Malmquist–Luenberger index to break down tourism eco-efficiency. The spatial variation function and GWR analysis describe its spatiotemporal evolution characteristics and allow the integration of econometrics, spatial geography, and other disciplines.

### Management implications

Tourism eco-efficiency is an essential index for the formulation of a tourism development plan, the evaluation of tourism management, and the promotion of the sustainable development of the tourism destination. The evaluation of tourism eco-efficiency, the description of the time evolution path, the outline of the spatial distribution pattern, and the discussion of its dynamic correlation with the development of the tourism economy have significant practical value. In the face of increasing tourism energy consumption and the worsening of the ecological environment, Inner Mongolia tourism eco-efficiency evaluation can effectively reflect the relationship between economic activities and the ecological environment. This is validated by the development of the worsening situation of the tourism ecological environment, can boost sustainable blossom of the province tourism such as national macro policy takes root provides the beneficial reference. The exploration of the time evolution model and the outline of the spatial pattern of tourism eco-efficiency in Inner Mongolia elucidate the sustainable development of the entire region's tourism in Inner Mongolia from a macro perspective and provide scientific guidance for the “top-down” decision and “bottom-up” policy of tourism ecological protection in Inner Mongolia. Moreover, this study explores the spatial and temporal patterns of tourism eco-efficiency and its influencing factors in Inner Mongolia, which provides a reference for the optimization of tourism eco-efficiency and the sustainable development of tourism in other regions.

Under the national macro context of constructing an ecological civilization, Inner Mongolia should change its tourism development model, enhance tourism eco-efficiency, and promote the sustainable development of tourism. Therefore, this study proposes the following. In view of the performance of tourism eco-efficiency in Inner Mongolia, the environmental ecology of tourism in Inner Mongolia needs to be improved, and environmental protection needs to be considered while developing the tourism industry, to enhance the sustainable development of tourism (17, 18). First, the government can stipulate relevant laws and regulations, formulate the ecological red line for tourism development, limit carbon emissions from tourism-related enterprises, and reduce environmental pollution from tourism activities. We need to focus on changing the tourism development model and enhancing tourism eco-efficiency in Inner Mongolia. The black linear development mode characterized by high energy consumption, high pollution, and low income should be gradually discontinued, and a green circular development characterized by low consumption, low pollution, and high income should be formed (49). Second, tourism eco-efficiency among cities in Inner Mongolia was observed to be heterogenous; therefore, targeted development countermeasures and suggestions based on local conditions are required to strengthen their cooperation, learn from each other's advanced experience, take the resource-efficient and environmental-friendly tourism development path, and jointly promote the sustainable development of tourism in Inner Mongolia. Inner Mongolia should continue to strengthen intra-provincial cooperation and actively draw on advanced experience from other provinces, adhere to the basic principle of strengthening external cooperation and internal communication, promote the transformation of the tourism development mode, and improve tourism eco-efficiency through knowledge and technology spillover as well as capital and talent radiation (50). Finally, the tourism development strategy should be constantly adjusted to promote the two-way synergistic improvement of the tourism economy and tourism ecology. As the global economy enters a new normal, the blind pursuit of tourism industry expansion should be abandoned, and the continuous improvement of tourism development quality should be promoted, in order to provide the funds, talents, information, and technology required to ensure tourism ecological protection.

### Limitations

This study explores in depth the spatial pattern and influencing factors of tourism eco-efficiency in Inner Mongolia. The study provides a reference for future research on tourism eco-efficiency and sustainability in Inner Mongolia and other regions. However, this study has certain shortcomings that should be addressed. First, regarding the design of the index system, carbon emissions as an undesirable output of tourism eco-efficiency reflect the negative impact of tourism on the environment. However, related theoretical and empirical research is insufficient; the travel coefficient of carbon emissions for Inner Mongolia has not been evaluated. Therefore, the measurement results of tourism eco-efficiency may be slightly conservative (21, 22). Secondly, given the ease of access to data, this study analyzes tourism eco-efficiency in a single province of Inner Mongolia, which can reflect the actual development status of tourism eco-efficiency. In future research, the perspective can be extended to a large-scale analysis of the region and the entire country (32, 33). In addition, this study analyzes the spatial layout and influencing factors of tourism eco-efficiency in the tourist attractions of Inner Mongolia and proposes recommendations for the optimization of their spatial structure. Therefore, future research should deeply analyze the reasons for the unreasonable spatial structure of tourism eco-efficiency, and should propose reasonable recommendations for the optimization of the tourism eco-efficiency structure, for the improvement of tourism eco-efficiency in similar areas (51).

## Conclusion

This study first constructs the tourism eco-efficiency evaluation index system and then explores the evolution path and spatial pattern of tourism eco-efficiency in Inner Mongolia from the perspective of the geographic spatiotemporal dimension. Inner Mongolia has a relatively low tourism eco-efficiency value, with an average value of 0.74. Furthermore, the tourism efficiency values of the provinces vary and their distribution is unbalanced. The range parameters of tourism eco-efficiency and the spatial correlation effect of tourism eco-efficiency in Inner Mongolia under the influence of structural and spatial differentiation showed a decreasing trend.

## Data availability statement

The original contributions presented in the study are included in the article/supplementary material, further inquiries can be directed to the corresponding author.

## Author contributions

XW contributed to all aspects of this work. YW wrote the main manuscript text and analyzed the data. Both authors reviewed this manuscript, read, and agreed to the published version of the manuscript.
